# Use, Risk and Revalorization of Veterinary Antibiotics: A Canadian Perspective

**DOI:** 10.3390/antibiotics14070665

**Published:** 2025-06-30

**Authors:** Laurence Auger, Linda Saucier, Marie-Lou Gaucher, Grant W. Vandenberg, Antony T. Vincent, Alexandre Thibodeau, Marie-Hélène Deschamps

**Affiliations:** 1 Département des Sciences Animales, Faculté des Sciences de L’Agriculture et de L’Alimentation, Université Laval, Québec, QC G1V 0A6, Canada; laurence.auger.1@ulaval.ca (L.A.); linda.saucier@fsaa.ulaval.ca (L.S.); grant.vandenberg@fsaa.ulaval.ca (G.W.V.); antony.vincent@fsaa.ulaval.ca (A.T.V.); 2 Chaire de Recherche en Salubrité des Viandes (CRSV), Département de Pathologie et Microbiologie, Faculté de Médecine Vétérinaire, Université de Montréal, Saint-Hyacinthe, QC J25 2M2, Canada; marie-lou.gaucher@umontreal.ca (M.-L.G.); alexandre.thibodeau@umontreal.ca (A.T.)

**Keywords:** animal production, veterinary antibiotics, Canadian livestock, antibiotic resistance, environmental impact, waste management, upcycling, insects as food, feed and fertilizers

## Abstract

The extensive use of veterinary antibiotics in livestock production is a growing concern, particularly in terms of environmental sustainability and health security. This review presents the case of veterinary antibiotic use and regulations in Canada before exploring a potential novel avenue for agricultural antibiotics waste up-cycling. The impact of the widespread use of antibiotics in animal husbandry is reviewed, and the dissemination routes of antibiotic residues and antibiotic-resistant bacteria from farms to the environment are explored to identify potential weaknesses in the management of veterinary antibiotics. The presence of antibiotic residues in livestock products and manure poses significant challenges, as these residues contribute to the development of antibiotic-resistant bacteria, which poses a threat to both the environment and health. The review examines the fate of animal waste contaminated with antibiotics in the environment, exploring the impact of management practices on antibiotic degradation and their persistence in soil and water systems. Additionally, the potential risks to human and animal health are addressed, emphasizing the links between antibiotic residues in the environment and the rising threat of antimicrobial resistance. The last part of this review focuses on exploring how up-cycling veterinary antibiotic residues in insects for feed and fertilizers could contribute to mitigating these risks. Overall, this review calls for more integrated solutions that balance the need for antibiotics in animal agriculture with the prevention of environmental contamination and the antibiotic resistance threat, while meeting the rising demand for animal proteins, highlighting the need for more region-specific surveillance programs.

## 1. Introduction

Since their discovery, the use of antibiotics has become widespread across the world. Antibiotic consumption rose by 65% between 2000 and 2015, reaching a total global consumption of 36,000 metric tons, and had a further increase of 21% between 2016 and 2023 [1]. In 2020, Canada was ranked as the fourth largest consumer of antimicrobial active ingredients in production animals (per kilogram of animal) among the 29 countries participating in the European Surveillance of Antimicrobial Consumption [2].

Worldwide, antibiotics play an indispensable role in modern health, livestock production, and economic stability, by preventing and treating infections in humans and animals as well as safeguarding food security, thereby contributing significantly to health and the economy. While antibiotics are essential tools for promoting health and productivity, their use also contributes to the growing challenge of antimicrobial resistance. The misuse and overuse of these drugs in livestock, such as industrial-scale usage for growth promotion that was common until recently, contributed to the increase in resistant bacterial strains [3]. This not only jeopardizes animal health and agrifood production but also has implications for human health and environmental ecosystems. In the last few years, the importance and multidisciplinary aspects associated with antibiotic usage have been put forward by the One Health framework [4]. A comprehensive understanding of veterinary antibiotic usage patterns is crucial to integrate human, animal, economic, and environmental facets. In this review, we describe antibiotics and their role in the antibiotic resistance problem, the status of antibiotics used in Canadian livestock, and the current determining factors in antibiotic-resistance ecology associated with common practices. We utilize the Canadian situation to explore the regional differences in veterinary antibiotics usage, highlighting the differences between the trends in their national and provincial context. This emphasizes the need for surveillance programs tailored to the Canadian landscape to guide evidence-based policies and stewardship programs for more efficient regulatory frameworks. Furthermore, we discuss an innovative up-cycling avenue to guide the development of new strategies to reduce the risks of veterinary antibiotics use in livestock.

## 2. Antibiotic Mechanisms

Antimicrobials are a diverse category of therapeutic agents encompassing a wide range of substances used in human and veterinary medicine, as well as in agriculture, for their efficacy in targeting microorganisms (bacteria, fungi, viruses, and parasites) [5]. The term antibiotic refers specifically to the class of antimicrobials that act against bacteria, but some antibiotics can also be used against parasites [6]. Antibiotics are regarded as one of the most significant medical discoveries of the 20th century [7]. Antibiotics are natural compounds synthesized by microorganisms, notably bacteria from the Actinomycetota phylum and filamentous fungi. Semi-synthetic antibiotics are chemically altered natural antibiotics modified to enhance their efficacy, stability or spectrum of activity. For instance, methicillin is a semi-synthetic antibiotic derivative of penicillin, designed to increase its stability and resistance to degradation by the enzyme penicillinase. The limited number of naturally occurring antibiotics discovered lately led to the development of fully synthetic antibiotics, such as sulfonamides and quinolones [8].

The mechanisms of action of antibiotics differ based on the active compound and can exert either bactericidal or bacteriostatic effects by targeting specific processes essential for bacterial cell survival or function. Antibiotics are categorized based on inhibition target: DNA/RNA synthesis, protein synthesis, cell wall synthesis, and folic acid metabolism [9]. Antibiotics are also grouped by structural resemblance into classes, even though antibiotics belonging to the same class can have different pharmacology. Each antibiotic has a spectrum of activity, from narrow to broad, affecting multiple bacteria. To exert their bactericidal or bacteriostatic effects, the active ingredient of an antibiotic must bind to a specific site (“active site”) of the bacteria. Additionally, the antibiotic must be present in sufficient concentration to bind to a critical number of these specific sites and remain bound for an adequate duration [10]. An antibiotic given at a lower concentration will change from a bactericidal effect to a bacteriostatic effect. The relationship between the concentration of an antibiotic and its binding to active sites is represented by the area under the concentration-time curve (Cp × time = AUC). Since this value (AUC) is typically not directly measurable, blood concentration is used as a proxy. To determine the appropriate antibiotic dosage, the blood AUC is compared to the minimum inhibitory concentration (MIC) of the bacteria. The parameter AUC/MIC indicates the extent to which the concentration and exposure time of the antibiotic exceed the minimum required for its bactericidal or bacteriostatic activity. A higher AUC/MIC ratio is associated with a greater likelihood of effectively eradicating the bacteria. Generally, oral administration of the antibiotic induces therapeutic blood levels similar to injection [5]. The extent and rate at which the active ingredient of the antibiotic enters the blood (and therefore the action site) is defined by bioavailability. The bioavailability of an antibiotic administered orally depends on the species and is generally relatively low, ranging from 1% to 20%. Therefore, an important quantity of the antibiotic initially administered will be excreted. For example, ciproflaxin has a good bioavailability in humans, but only a fair to negligible bioavailability in dogs and horses [5,11]. Antimicrobials can either be water-soluble, their distribution being limited to extracellular fluid (i.e., beta-lactams, fosfomycin, aminoglycosides, some sulfonamides and tetracyclines), or lipid-soluble, enabling them to reach intracellular organisms and the potential for bioaccumulation at specific sites (i.e., fluoroquinolones, macrolides, clindamycin, many sulfonamides, doxycycline and minocycline) [5].

## 3. The Ubiquitous Use of Antibiotics and Antibiotic Resistance

Since their discovery, antibiotics have become commonly used in healthcare and agrifood systems, leading to a significant dependency of contemporary systems. Antibiotics are considered the most effective weapon against pathogenic bacteria in both human and veterinary medicine. It is estimated that in 2018 alone, antibiotics contributed to saving 17,000 human lives and preventing 2.6 million days of hospitalization in Canada [12]. In low and middle-income countries, antibiotics have also contributed to reducing the mortality of children under five years old from 21.6% to 3.5% between 1950 and 2017 [13].

Antibiotics are the primary therapeutic tool for treating severe bacterial infections in human health (i.e., infections caused by harmful bacteria that can lead to severe illness or complications if left untreated). However, their use exerts selective pressure on bacteria, accelerating the emergence of antibiotic resistance genes (ARGs) [14]. The increase in antibiotic resistance among pathogenic bacteria threatens the availability of effective treatments for serious bacterial infections. The loss of antibiotic efficacy due to resistance is occurring more rapidly than the development of new alternative drugs, making bacterial resistance a major global issue affecting health, food, environment, and economics [15]. Antibiotic resistance in pathogenic bacteria leads to longer and more severe illnesses, increased risk of disease transmission, higher healthcare costs, elevated mortality risks, and financial losses in animal production. The decline in antibiotic effectiveness places additional stress on an already strained healthcare system and could profoundly alter current healthcare practices. In Canada, approximately 26% of bacterial infections are resistant to antibiotics, directly responsible for over 5400 deaths in 2018 and resulting in an estimated economic impact of CAN$ 2 billion on the country’s GDP [12]. Resistance rates for first-line antimicrobials (i.e., drugs that are used as the optimal first choice of treatment for infections) are projected to reach 40%, potentially leading to 39,600 annual deaths from resistant bacterial infections and a reduction in Canadian GDP by up to CAN$ 21 billion per year [12].

The agrifood industry is the largest consumer of antibacterials. While the demand for animal proteins is growing worldwide, antimicrobials are a key component to prevent the spread of infections in farm animals, ensuring animals’ rights to welfare and maintaining the actual level of productivity to meet consumer expectations [16]. The practice of using antibiotics in animal production for both therapeutic and prophylactic purposes has been firmly established worldwide for decades [17]. In 2017, 73% of antimicrobials used worldwide were intended for animals [18]. In 2020, in Canada, 82% of antibiotics were used for animal husbandry, representing a quantity (mg/PCU) three times higher than the median reported in the European Union [19,20]. The use of antibiotics varies across agrifood sectors, with the highest consumption occurring in livestock production. According to the Canadian Animal Health Institute, “[The use of antibiotics] can represent a responsible and safe way to enhance the health and well-being of animals.”, mainly by curing and preventing diseases (https://cahi-icsa.ca/animal-health-industry/use-of-antibiotics-and-hormones-in-food-animals, accessed on 11 March 2025). In Canada, animals have a right to welfare, meaning there is a responsibility to cure and reduce suffering, which applies indiscriminately to pets and livestock.

Plant pathologists also recognized the potential of antibiotics to control bacterial diseases in agriculture, progressively replacing since the 1950s metal-based bactericides that were needed in high dosages, associated with a toxic effect on plants [21]. However, compared to animals, plants account for a negligible amount of antibiotic utilization (~0.5% in the USA) [21].

Resistance to antibiotics is a natural result of the evolutionary arms race between organisms [15]. Most of the antibiotics used today existed in the environment long before we discovered them, with bacteria inhabiting these environments and having to adapt to these and all kinds of xenobiotics. However, industrial production of antibiotics and their widespread use are the primary factors driving the increasing rate of bacterial resistance [14]. Moreover, the limited diversity of available antibiotics means that some antibiotics critical for human health are also used in animal production and agriculture, thereby increasing the risk of selecting ARGs in pathogenic bacteria that affect humans [15].

Antibiotics are ubiquitously employed in human health, agriculture, horticulture, and aquaculture, influencing the prevalence of resistant bacteria. Unnecessary use and misuse of antibiotics are recognized as avoidable contributors to the accelerated selection of ARGs within human, animal, and environmental bacterial populations. According to the Centers for Disease Control and Prevention of the United States (CDC), unnecessary use of antibiotics is defined as the use of antibiotics when they are not needed, and misuse is when the antibiotic, dosage or duration of treatment is wrongly used (https://www.cdc.gov, accessed on 11 March 2025). Mechanisms of antibiotic resistance can be intrinsic or acquired and include the protection or substitution of the antibiotic target, the detoxification of the antibiotic and the blockage of antibiotic intracellular accumulation [22].

**Figure 1:**
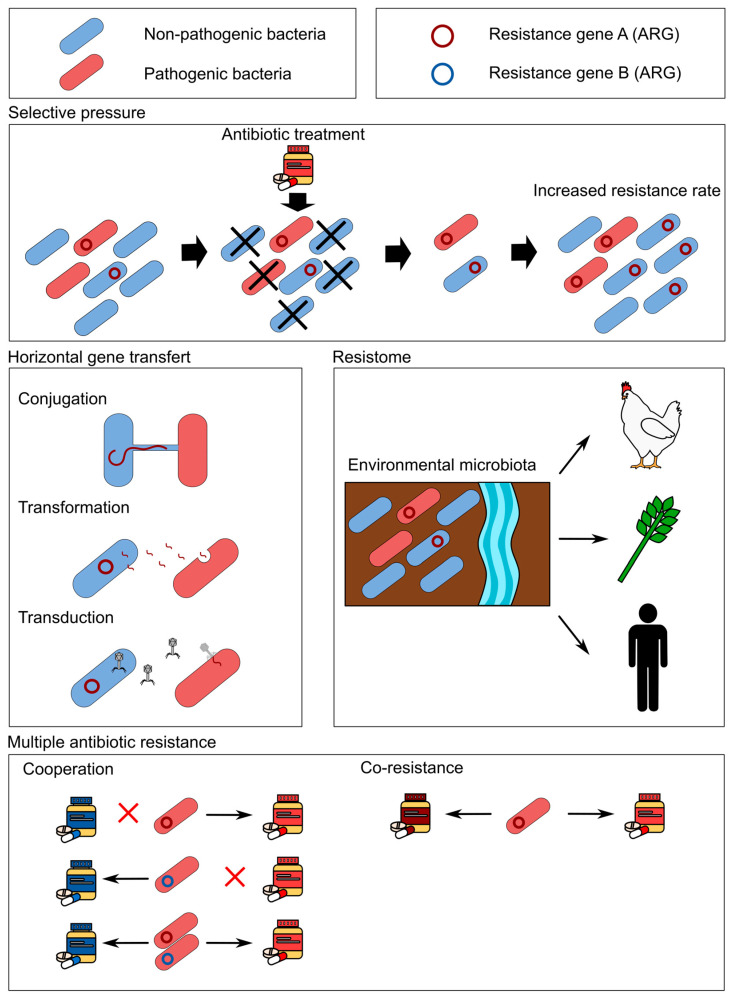
Mechanisms of ARGs selection, transfer and resistance.

Pathogenic bacteria are not the only microorganisms of concern in the antibiotic resistance problem. The presence of ARGs in commensal bacteria also exacerbates health issues, as these bacteria serve as reservoirs for ARGs that can be transferred to pathogenic bacteria through horizontal gene transfer mechanisms [23]. Horizontal gene transfer enables bacteria to exchange genetic material (i.e., plasmids, transposons and integrons) among themselves during their lifecycle, by mechanisms such as conjugation, transformation and transduction (Figure 1) [24]. As a result, a sensitive bacterium can acquire genes conferring resistance to one or more antibiotics [25]. The acquisition of ARGs can occur even between phylogenetically distant bacteria [26]. For example, plant pathogens were found to have acquired streptomycin-resistance genes (*strA-strB*) from nonpathogenic plant epiphytes and soilborne bacteria in orchards where the antibiotic was used [21]. Human pathogens’ resistance genes originate from diverse bacterial sources, showing the importance of monitoring all bacteria as a single global gene pool that provides tools for resistance across species [22]. Even the natural gut microbiota can become the reservoir of antimicrobial resistance genes, like the IncL broad-host-range plasmids that often encode the carbapenemase OXA-48 (enzymes able to degrade carbapenem antibiotics), which was observed to be readily transmitted through conjugation between the pathogen *Klebsiella pneumoniae* and members of the gut microbiota in patients [27].

Certain resistance genes may be linked and transferred simultaneously, providing multiple antimicrobial resistance (co-resistance). These linked genes are frequently found in larger genetic elements such as integrons, transposons and resistance plasmids—extrachromosomal, self-replicating genetic elements that can encode from a few to several hundred genes [25]. The concurrent transfer of multiple linked ARGs exacerbates the issue of antibiotic resistance by facilitating the selection of multidrug-resistant bacteria [28]. Furthermore, the selective pressure exerted by an antibiotic on an ARG may contribute to the co-selection of co-resistance [29]. Since the 1970s, a strain of *Mycobacterium tuberculosis* has gained co-resistance, including isoniazid, streptomycin and para-aminosalicylic acid, proving to be a major medical challenge. Furthermore, a given ARG may provide resistance to multiple antimicrobial agents of the same class (cross-resistance) or of different classes (cross-class resistance) [29,30]. One antibiotic resistance mechanism is the active transport of molecules outside of the cell membrane by multidrug efflux pumps [31].

These multidrug efflux pumps with polyspecificity for a diverse range of compounds, like the AcrAB-TolC pump, which are common to many Gram-negative bacteria (e.g., *Escherichia coli, K. pneumoniae, Pseudomonas aeruginosa*), provide decreased susceptibility to antibiotics like oxacillin, rifampicin, enoxacin, tetracycline, etc. [32]. Bacteria that have acquired resistance to antimicrobial agents from at least three categories are defined as multidrug-resistant (MDR) bacteria. These antimicrobial categories are based on chemical relations between antimicrobials, meaning they share the same action target [33]. Bacteria resistant to a given agent in a class will generally also be resistant to the other agents of the same class (see Table 1). Bacteria that are sensitive to an antibiotic may be protected from the antibiotic treatment through the resistance mechanism or degradation activity of the antibiotic by other bacteria present in the same environment [34]. This type of cooperation is often associated with β-lactam, bacteria that secrete β-lactamase enzyme degrading antibiotics [35].

In the late 1940s, the biologist Thomas Jukes was the first to propose that antibiotics could be utilized in animal feed to improve performance, leading to a revolution in industrial agriculture with the adoption of antibiotics as growth promoters [37]. This widespread practice consisted in administering antibiotics at lower concentrations than those used for prophylaxis or treatment, which increased the rate of biomass gain in many farm animals. Growing concern over the rapidly increasing rate of antibiotic resistance led the World Health Organization (WHO) to recommend in 1997 that antibiotic use as growth promoters be banned. In 2006, the European Union addressed the growing issue of ARGs by prohibiting the use of antibiotics as growth promoters (Regulation 1831/2003/EC).

The biggest concern of veterinary antibiotics usage is their role in the transmission of antimicrobial resistant (AMR) pathogens to humans. The most direct route of transmission of antibiotic-resistant bacteria from livestock to humans is through the contamination of meat. The risk of meat contamination by antibiotic resistant bacteria is positively correlated with the use of antibiotics in animal production [38]. Multiple measures have been put in place to ensure the microbiological safety of meats, such as feed withdrawal and antibiotic withdrawal before slaughter. Nevertheless, raw meat may contain antibiotic resistant microorganisms [39]. Another avenue of direct transmission is from the close contact between producers and their livestock. Indeed, producers are reported to be more likely to be infected with methicillin-resistant *Staphylococcus aureus* than other individuals [40]. Slaughterhouse workers were also found to have an increased rate of ARGs in their feces, correlated with the amount of time spent working [41]. Indirect transmission may occur from the dispersion of antibiotics or ARGs in the environment, notably by the contamination of agricultural soils. For example, crops cultivated in soil fertilized with manure or wastewater contain more ARGs than crops without these additives, and these ARGs could be transmitted to consumers [42]. Antibiotic resistant bacteria are found to be more abundant in the environment in proximity to the farm, suggesting this transmission route only applies at the local scale [43,44].

Reducing the usage of medically important antibiotics is the main recommended strategy to address antibiotic resistance. It is essential to implement measures that limit the use of medically important antibiotics, especially those with few or no alternative treatments for severe bacterial infections. Combating antibiotic resistance requires a comprehensive and coordinated approach that is implemented simultaneously across all sectors where antibiotics are used. This holistic view of the antibiotic resistance problem is reflected by the “One Health approach”, recognizing that human, animal and ecosystem health are interdependent. This approach promotes “collaborative, multisectoral and transdisciplinary” actions to achieve optimal health outcomes [12]. To address the issue of bacterial antibiotic resistance, it is essential to use antibiotics judiciously, meaning using antibiotics responsibly and appropriately across human, animal and environmental health sectors. This entails reducing their use, ensuring adequate monitoring, preventing infections, and investing in alternatives to mitigate their impact at the human, animal and environmental levels.

## 4. The Canadian Regulation and Surveillance of Veterinary Antibiotics Use in Livestock

In Canada, the regulation of veterinary antibiotic use is overseen by a range of entities, including Health Canada and the Veterinary Drugs Directorate (VDD) at the federal level, as well as provincial and territories organizations. The federal government emits regulations on veterinary drugs import, approval, packaging and labeling. Although not exhaustive, the class categories for the antimicrobials distributed for sale in Canada are presented in Table 1. Provincial governments play an important role in overseeing the distribution and administration of veterinary medicines, relying on veterinarians’ official professional organizations for enforcement.

Steps were taken in 2018 to reduce the usage of antibiotics by removing the growth promotion and production enhancement claims from labels of medically important drugs. The same year, a regulation amendment mandated veterinary prescriptions for the use of medically important antimicrobials in animal production. However, no specific regulations outright prohibit the use of antimicrobials for growth stimulation and increased feed efficiency in Canadian livestock [45]. Continued use of medically important antimicrobials as growth promoters was still reported in 2020 for four flocks of broiler chickens or turkeys [19]. As of 2024, growth promotion remained a listed claim for the use in class IV antimicrobial substances in animal production on the Canadian Food Inspection Agency (CFIA) website (https://inspection.canada.ca/en/animal-health/livestock-feeds/medicating-ingredients, accessed on 11 March 2025).

The CFIA classifies antibiotics into four distinct categories based on their level of importance for human health, ranging from Category I (very high importance) to Category IV (low importance; see Table 2). These categories are organized according to whether the antibiotic is considered a preferred treatment option for serious infections in humans (resulting in significant morbidity if left untreated) and the availability or rarity of alternative medications. However, the use of antibiotics of low importance for human health may indirectly increase the resistance of bacteria to antibiotics used in human medicine because of cross-resistance. This happened with ceftiofur, a cephalosporin antimicrobial that was not used in human medicine and therefore was not considered to be of human medical importance, which was commonly employed in hatcheries for the prevention of early chick mortality. The bacteria *Salmonella* can become resistant to ceftiofur by producing β-lactamase enzymes that break down the β-lactam ring structure of the antibiotic, a structure essential to the antibiotic action. However, this resistance mechanism can also target other cephalosporins because of their structural similarity. The use of ceftiofur therefore induced the cross-resistance of *Salmonella* to ceftriaxone, an important cephalosporine antibiotic in human medicine used to treat severe infections caused by *Salmonella* [46,47].

In Canada, the national integrated surveillance of antibiotic use is managed by the Canadian Integrated Program for Antimicrobial Resistance Surveillance (CIPARS) of the Public Health Agency of Canada. Utilizing data provided by federal, provincial, and private industry partners, CIPARS collects, analyzes, and communicates trends in antimicrobial use and antimicrobial resistance. The antibiotics listed in the Canadian compendium of medicating ingredients and their recommended dosage for veterinary use are presented in Table 3.

New regulations established in 2017 ensure monitoring of the annual sales of medically important antimicrobials for animal productions. Since 2018, manufacturers, importers, and preparers have been required to report all sales of antibiotics. The CIPARS reports on data collected through the Veterinary Antimicrobial Sales Reporting (VASR). It acts as a surveillance tool, providing an overview of antibiotic sales for veterinary use in Canada [20]. However, it should be interpreted with caution as it does not reveal actual usage trends. It is also important to note that AVDPRS data excludes Category IV antimicrobials. Furthermore, CIPARS data offer limited flexibility for in-depth analysis, lacking the capability to differentiate usage details by provinces and territories. Given the variation in sector predominance, government programs, regulations, and policies across provinces, a comprehensive understanding of veterinary antibiotics usage trends within each province is needed to inform more targeted regulatory frameworks and identify regional variations in practices. It is currently challenging to obtain a representative picture of the situation in each province, as little data is available on province-specific usage and trends.

In 2022, Ontario was the largest seller of medically important antimicrobials in Canada (30.4%), followed by Alberta (25.4%) and Quebec (23.0%). Of the total medically important antibiotics sold in Canada in 2022, the vast majority (92%) were intended for animal feed, including as pre-mixed feed (75%), in water (16%), or administered orally (1%).

A total of 1,005,157 kg medically important antimicrobials were sold for animal uses (pets and livestock), with the majority (675,096 kg, or 67.2%) classified as Category III. The most sold antibiotics were tetracyclines (510,733 kg, Category III), macrolides (105,038 kg, Category II), penicillin (97,969 kg, Category II), sulfonamides (62,897 kg, Category III), and lacosamide (51,753 kg, Category II). The swine industry is by far the largest consumer of antimicrobials (539,276 kg, or 53.7%), followed by cattle (311,120 kg, or 31.0%) and poultry (99,188 kg, or 9.9%). Despite its leading consumption of antibiotics, the swine sector does not differ significantly from the cattle sector in the number of animals raised: in 2022, there were 14.1 million pigs compared to 12.6 million cattle [49]. Consequently, swine production in Canada was the sector with the highest consumption of medically important antimicrobials relative to biomass produced (297 mg/PCU), followed by poultry production (197 mg/PCU). In swine production, the three primary classes of antimicrobials sold were tetracyclines, penicillin, and macrolides [20].

A component of the CIPARS program is the CIPARS-FARM, specialized in the collection of data from 272 volunteer sentinel farms (115 broiler chicken, 61 turkey, and 96 swine finishing operations). It is the only surveillance program that provides the visualization of data based on components such as type of livestock, class of antimicrobials, route of administration, antimicrobial categorization, and region (national or provincial). Data on Category IV antimicrobials are not included. However, the limited farm sample means the data trends may significantly differ from the global animal production landscape. Nevertheless, these data offer valuable insights into the use of medically important antimicrobials within the context of each province and territory.

In 2023, the Pan-Canadian Action Plan on Antimicrobial Resistance was implemented as a collaborative work between federal, provincial, and territorial governments, with 10 key aspects, including the improvement of antimicrobial use monitoring. This program will become critical in understanding province-specific veterinary antibiotic usage.

## 5. Discharge of ARGs and ARM from Animal Production

While Canada’s regulatory framework for veterinary antibiotics aims to reduce their usage and mitigate the risks associated with their application, antibiotics remain an essential part of agricultural productions, which may lead to their discharge in the environment through various pathways. Most antibiotics used in animal production are administered orally (through feed or water), which exerts selective pressure on the gastrointestinal microbiota. This pressure facilitates the exchange of genetic material and the selection of ARGs [50]. Antibiotic-resistant bacteria are also known to be transmitted from treated animals to untreated animals living on the same farm [39]. It is commonly reported in the literature that animals absorb only a small percentage of most administered veterinary antibiotics, with a significant portion of these antibiotics being excreted in their active form directly in urine and feces [51,52,53]. However, few studies have investigated the actual fraction of antibiotics that is excreted by livestock (see Table 4). Even if the exact fraction and rate of excretion of antibiotics are unknown, multiple studies have reported their presence in high concentration in manure and other excreta, and alerted that antibiotic compounds can accumulate in soil [54,55]. The antibiotic-resistant bacteria, their ARGs, and active antibiotic ingredients issue from the use of antibiotics in animal production can be discharged in the environment through the use of manure and wastewater as fertilizers, contributing to the global health issue of antibiotic resistance [56].

The vast majority of animal excreta (>90%) is used as natural fertilizer in agriculture, because spreading is a conventional method for recycling nutrients in cultivated soils and provides a cost-effective waste disposal solution [57]. Antibiotics and ARGs dissemination in the environment can also occur through crop irrigation practices. The downside of spreading manure and slurry contaminated with antibiotics is the introduction of these antibiotic residues into the soil and watercourses, thereby increasing human exposure to antibiotics [55]. These antibiotics and associated resistant bacteria can subsequently be dispersed over large distances through aerosolization, animal movement, and water flow [58].

The indigenous bacteria present in soils naturally produce small amounts of antibiotics, generally under detectable levels. However, the concentration of these compounds in the environment could increase to problematic levels because of the considerable amount of manure spread in some locations. Indeed, the intensification of livestock production has dramatically increased the concentration of animals and created an overabundance of manure for the available area. In China, animal manure production was reported to be 2.17 billion tons annually in 2004, and in the United States, it was 1.1 billion tons in 2013 [52,59]. The concentration in soils of antibiotics like tetracycline seems to be consistently reported below the detection threshold outside of manure application [60].

Manure spread and irrigation with wastewater from animal production also present an ecotoxicological effect. The contamination of the environment by high concentrations of antibiotics can disrupt ecosystems through the alteration of the structure and activity of microbial communities, including their composition, diversity, abundance, enzyme activity, and ability to metabolize different carbon sources [61]. These natural communities play a crucial role in ecological systems, particularly in biogeochemical cycles and the degradation of exogenous compounds. Thus, antibiotic pollution can lead to significant ecological disturbances. Environmental bacteria, which colonize watercourses and soil, serve as reservoirs for ARGs, collectively known as the “resistome”, that can contribute to the transmission of ARGs to other bacteria they come in contact with, such as pathogenic bacteria [62].

Environmental contamination by antibiotics, along with the spreading of antibiotic-contaminated materials, poses a risk of accumulation in plant tissues and the selection of antibiotic-resistant phytopathogens in agricultural production [63]. For instance, the use of oxytetracycline to control *Xanthomonas arboricola* pv. *pruni,* which causes bacterial spot on plums and nectarines, has led to the selection of antibiotic-resistant phytopathogenic bacteria [64]. Additionally, some antibiotics exhibit phytotoxic effects, which are detrimental to plant growth [65].

**Table 4:** Excretion fraction of veterinary antibiotics in animal production.

Antibiotic	Active Ingredient	Animal	Dosage	Excretion (%)	References
**Tetracyclines**	Tetracycline	Pigs	Not reported	25–80	Feinman 1978 as cited by [66,67]
	Chlortetracycline		40 mg per kg bw	65–75	Veterinrermedicinsk Produktkatalog 1999, as cited by [68,69]
	Oxytetracycline		5–25 mg per kg bw	23–65	[70]; Veterinrermedicinsk Produktkatalog 1999, as cited by [68]
**Lincosamides**	Lincomycin		Not reported	60	Aiello 1998 as cited by [51]
**Macrolides**	Tylosin	Pigs & cattle	5–10 mg per kg bw	40–100	Feinman 1978 as cited by [66]; Veterinrermedicinsk Produktkatalog 1999 as cited by [68]
**Sulfonamide**	Metronidazole		Not reported	40	Kümmerer et al. 2000 as cited by [51]
	Sulfadiazine	Pigs	200 mg per 15 kg bw	90	Veterinrermedicinsk Produktkatalog 1999 as cited by [68]
	Sulfatroxazole	Cattle	200 mg per 15 kg bw	90	
	Sulfadoxine		200 mg per 15 kg bw	90	
	Sulfapyrazole		50–70 mg per kg bw	90	
	Chloroquine		Not reported	70	Goldsmith 1992 as cited by [51]
**β-lactam**	Penicillin G	Pigs	10–20 mg per kg bw	90	Veterinrermedicinsk Produktkatalog 1999 as cited by [68]
	Amoxicillin	Cattle	10–30 mg per kg bw	90	
	Ampicillin		15–30 mg per kg bw	90	
**Quinolones**	Enrofloxacin	Pigs & cattle	5–10 mg per kg bw	35	Veterinrermedicinsk Produktkatalog 1999 as cited by [68]
	Norfloxacin		Not reported	30	

## 6. Fate of Antibiotic Contaminants from Animal Production

### 6.1. Degradation in Soil

The fate of antibiotics in the environment depends on multiple factors, including the stability of the compound as well as the environmental conditions (e.g., temperature, light, matrix composition, pH, water content, etc.) [60,71]. Because antibiotics can vary widely in their physiochemical properties and their molecular structure, it is unsurprising that antibiotics in the environment will range from rapid degradation to persistence. Antibiotics may be degraded by both biotic and abiotic processes. In the environment, their degradation mainly occurs because of photodegradation in natural water and surface soil [72]. Some antibiotics were also found to be susceptible to hydrolysis and thermolysis, but biodegradation (complete mineralization of the compound by biological processes) appeared to be poor for most of the antibiotics investigated in laboratory settings [60]. The degradation of antibiotics produces secondary metabolites, which may still have antibacterial activity and, in some cases, even more potent activity than the parent compound. Some metabolites may also be hard to mineralize. The current knowledge of the degradation pathways differs with the studied antibiotic.

The degradation rate of antibiotics in soil differs widely, from a half-life of a few hours (e.g., amoxicillin) to a couple of years (e.g., tetracycline) [61]. Some persistent antibiotics with slow degradation rates in soil include fluoroquinolones, macrolides and tetracyclines. Antibiotics such as tetracyclines tend to adsorb onto soil organic matter [73]. The binding of antibiotics to particles or the formation of complexes may lower or eliminate the antibacterial activity of the compound or interfere with detection methods (e.g., high-performance liquid chromatography coupled with mass spectrometry) [60]. For example, tetracycline’s main removal mechanism in activated sludge was determined to be sorption, as no biodegradation was observed [74]. The bioavailability and mobility of the antibiotics in the environment are influenced by the coexisting substances in the environment, the matrix and the environmental conditions [75]. These interactions of antibiotics with compounds in the environment may lead to an underestimation of their prevalence. For example, tetracycline antibiotics were found to create non-covalent bonds with proteins in chicken meat, preventing the detection of the compound in this matrix [76].

### 6.2. Effect of Manure Treatment on Antibiotics

Livestock waste does not require treatment prior to disposal, unlike human biosolid waste. On farms, animal waste is generally sequentially transferred to storage, stored for various periods of time, removed from storage, transported to land and applied on land with specialized machinery. Before spreading on land, organic materials must be stabilized through decomposition. In Canada, 69% of livestock waste is stored in solid or semi-solid form, 14% is stored as a liquid, and 24% is not stored [77].

Diverse practices can be used to manage or treat manure, including turning, actively composting, anaerobic digestion, or mixing with additives. While these practices could help reduce bacterial loads and antibacterial concentration in manure before application, 71% of Canadian dairy producers, 49% of beef producers and 71% of poultry producers did not treat waste by any means in 2021 [78]. In Quebec, fewer producers use treatment practices, with 80% of dairy producers and 78% of poultry producers who did not treat their solid manure in 2021 (beef producer data was unavailable for this reference period) [78]. Simple storage of animal residues before application does not reduce the abundance of resistant bacteria and ARGs present [79]. However, even when they are used, these conventional livestock waste treatments are not specifically designed to remove antibiotics and ARGs contaminants. Data on the treatment practices used by Quebec producers are unavailable, but in Ontario, 8% of beef producers actively composted and 32% “occasionally turned or mixed to encourage partial decomposition” of solid manure stored on the operation, while 7% and 21% of poultry farmers applied these respective treatments.

#### 6.2.1. Composting Effect on Antibiotics

The degradation of antibiotics during composting depends on both the active ingredient and the matrix of the compost [80]. For instance, in poultry manure, composting can reduce the presence of chlortetracycline by 90%. However, in pig manure, the antibiotic is only reduced by 27% by the end of the process [52]. Although composting can decrease the overall bacterial load, it does not effectively reduce the abundance of ARGs [81]. Even if compost can be efficient in degrading antibiotics, only 3% of beef cattle producers used this management system for manure in 2004 in Canada, and 0% for other animal types [82].

#### 6.2.2. Anaerobic Digestion on Antibiotics

Anaerobic digestion has received increased interest as a management tool for organic waste, mainly because it produces methane-rich biogas used as a renewable energy source. Numerous factors influence the efficiency of anaerobic digestion in degrading antibiotics, including the concentration of antibiotics, the bioreactor operating conditions, the matrix and the microorganisms communities [66]. These parameters also affect ARGs removal during the treatment [83]. Mesophilic anaerobic digestion systems reportedly reduce chlortetracycline by 80% and monensin (bacitracin) by up to 8% [84]. Thermophilic anaerobic digestion can have an increased effect on antibiotics reduction, up to 98% for chlortetracycline, 36% for clarithromycin and 90% for erythromycin; however, thermophilic systems also have a greater abundance of ARGs (24% more in cattle manure compared to mesophilic systems) [66].

Both composting and anaerobic digestion can contribute to the reduction of antibiotic contaminants and have mitigating effects on ARGs. However, Canadian farmers are foregoing these treatment applications and continue to spread untreated livestock waste. Perhaps because of concerns about the initial cost and investment, the complexity of management, the change in nutritional values of compost or for other reasons altogether. A better understanding of the factors that limit the use of organic waste treatment by producers is needed to put efficient action strategies in place to limit the antibiotic resistance risks, since this issue can only be tackled when coordinating all the actors involved. This highlights the importance of agricultural biowaste management strategies to prevent the contamination of the environment by antibiotics and ARGs.

## 7. A Revalorization Avenue for Antibiotic Contaminated Agricultural Waste

The common use of veterinary antibiotics in livestock is such an integral part of the current agricultural system of Canada (and of most developed countries) with no available alternative that there seems to be little choice but to continue their usage, despite the risk to human health. However, the dissemination of antimicrobials in the environment has a severe impact on ecosystems and is associated with the problem of antibiotic resistance in human health. Therefore, the elimination of antibiotics as contaminants is of great concern. Alternative avenues should be explored to minimize the impact of veterinary antibiotics use.

Since the publication of a report by the United Nations in 2013 on the use of insects as food and feed, industrial productions have been rapidly increasing. The primary value of some insects is their ability to convert low-value residual organic stream into high-value products, mainly quality proteins and lipids in the larval biomass, used in animal feed. A co-product of insect rearing is frass (i.e., residue of bioconversion composed of organic matter, chitin and insect excreta), sold as a biofertilizer. Insect production, subscribing to a circular economy model (using low-value organic resources) could offer a solution to reduce waste by upcycling while simultaneously answering the growing global demand for animal proteins, reducing the pressure on feed resources and lowering the environmental impact of livestock production [85]. Insects like the black soldier fly (*Hermetia illucens*) can be reared on a wide diversity of substrates, from vegetal to animal. Larvae can even be reared on manure and are proposed as a strategy to manage waste stream, lowering the water content and mass [86].

This approach, using insect technologies to upcycle antibiotic-contaminated wastes like manure, constitutes a form of revalorization by transforming residues that could pose biological risks into high-value biomass and fertilizer. However, this is dependent on the ability of the final products of the insect production (larval biomass and frass) to be of increased value compared to the initial product. If antibiotics are successfully biodegraded during the bioconversion process and final products are safe, larval biomass and frass both have a significantly higher economic and functional value than the initial waste. Larvae are a source of high-value proteins and lipid-rich biomass that is suitable for animal feed and aquaculture, but they can also have non-feed applications such as biogas production, pharmaceutical products and waste stream management [87,88]. Additionally, frass presents an increased nitrogen content and can have beneficial effects on plant health and productivity [89,90]. Concisely, insect technologies could not only neutralize the environmental liabilities of contaminated waste but also create final products that are marketable with agronomic, industrial and environmental benefits. There are limitations to black soldier fly technology, like the rearing environmental needs of this tropical species that can be cost-prohibitive at smaller production scales or for specific industrial models, especially in temperate regions like Canada. Furthermore, bioconversion performance is dependent on the nutrient quality of the rearing substrate [91]. A specific waste stream may also not cover all the nutritional requirements of the insects and would need to be combined with other nutrient sources to ensure the sustainability of the production.

The idea of using entomoremediation is a new field of research [92]. Even insects that are not industrially reared for organic waste bioconversion have been proposed to be used as industrial pollutants bioremediators, such as a *Drosophila melanogaster* engineered to express a fungal laccase [93]. Most of the research interest has been directed toward the use of the black soldier fly larvae to bioremediate contaminants, including inorganic (e.g., heavy metals) and organic pollutants in substrates [94]. The strategy for the removal of inorganic pollutants such as heavy metals is the bioaccumulation of these compounds in the larval biomass [95]. The resulting larva products are contaminated and cannot be sold for nutrition, but could be sent to other non-feed uses. However, this usage goes against the most common objective of larval industrial rearing, which is to generate high-quality products for animal feed markets. Black soldier fly larvae can bioaccumulate heavy metals such as cadmium, manganese and lead, depending on the rearing substrate composition [94,96,97]. Organic pollutants could potentially be removed by partial or complete degradation, without accumulating in the larval biomass. The resulting co-products (larvae and frass) would be exempt from pollutants and therefore compatible with the actual production goals of industrial rearing. However, to ensure the security of the final products for the alimentation and the environment, more studies are needed.

Some studies have explored some specific antibiotic bioremediation capabilities of the black soldier fly larvae during bioconversion. In 2020 and 2022, Liu et al. investigated the bioconversion of soybean meal spiked with oxytetracycline to determine the potential for the production of waste management of agricultural productions and the discarded bacterial residue from pharmaceutical factories’ antibiotic production [98,99]. Oxytetracycline was degraded by 69 to 83%, with an inverse relationship to degradation rate and initial concentration of the antibiotic (100 to 2000 mg/kg) in the substrate. Another study by Mei et al. found that the bioconversion by the larvae degraded tylosin and enrofloxacin in swine manure by 82% and 87%, respectively [100]. The larval gut microbiota was found to be a major contributor to the degradation, increasing by 40% the reduction in the antibiotics in the substrate. Larvae at the end of the bioconversion were exempt from tylosin, but not from enrofloxacin. The contribution of the larval gut microbiota was also observed in the degradation of ciprofloxacin in chicken manure [101]. The antibiotic was reduced by 84% in the course of 12 days. Another study on pharmaceutical industry wastes found that the larvae and their associated microbiota degraded lincomycin by 85% in lincomycin fermentation residues [102]. All these studies support that bioconversion by the larvae significantly increases the degradation of the antibiotics compared to natural processes.

While antibiotics degradation was successfully achieved in multiple studies, other parameters must be considered for the application of BSF technologies to degrade contaminated substrates. The presence of some of these veterinary compounds (oxytetracycline, eprinomectin and ivermectin) was found to reduce larval survival and/or weight [103,104]. While there are yet no reports of veterinary compound bioaccumulating in the larval body, transfer from the substrate to the larvae was observed. Notably, doxycycline added to the BSF rearing substrate at a concentration similar to contaminated manure was reported to be above the maximum residue level (MRL) of bovine muscle in the larvae [103]. Given that veterinary antimicrobials vary in their behavior and persistence, as well as the metabolites that they can produce, and that they are further modulated by environmental conditions, the fate of each of these compounds in the substrate and the larvae must be individually assessed before they can be integrated into BSF rearing substrates to accurately determine the safety of the final products. Antibiotics have also been shown to have growth-promoting effects in various insect species, similar to vertebrates [105]. A study on the tiger moth (*Arctia plantaginis*) suggests this may be the result of a shift in resource allocations to the detriment of immunity, which could have a significant impact on insect health and the biosafety of insect productions [106].

To date, no insect product from insect farming on animal waste has been accredited in Canada (Annex IV, CFIA). Nevertheless, the larval biomass produced by the treatment of these residues could be directed to other applications, such as chitosan, antimicrobial peptides, biodiesel and biogas production [95]. Black soldier fly reared for animal feed could still be used to upcycle pre-consumption residues contaminated with antibiotics. Because of its regulations, the Canadian market presents a significant potential for the application of BSF technology. In Canada, there are no regulations that categorically prevent the use of waste streams in insect feed. This contrasts with the European Union, which has a feed ban rule of Regulation (EC) prohibiting the inclusion of household waste, catering waste, meat-and-bone meal and manure in insect feed [107]. Ingredients for pet food have few restrictions, requiring only that they are produced in sanitary conditions. For animal feed, the producers must, however, ensure the innocuity of the final products and establish sanitary conditions for production. Standard risk management must be observed, and final products for animal feed must first be validated by the Canadian Food Inspection Agency. Insect ingredients for animal feed are regulated by the Feeds Act and Health of Animals Act. Currently, frass are not recognized as animal manure; their applicable framework is dependent on the type of feed intrants [108]. In Canada, the production of black soldier flies is mainly based on a mixture of non-meat pre-consumption organic residues. Milling residues (such as bran and barley) are typically added (approximately 20–30%) to control the moisture content of the residues (e.g., grocery residues with around 85% relative humidity), enhance their nutritional quality (proteins, lipids), and optimize the bioconversion process by the larvae. In some instances, the milling residues used could be redirected directly to animal consumption. Furthermore, their economic value is significant and is likely to increase in the future, potentially undermining the long-term profitability of production. Replacing these inputs with cheaper yet equally nutritious alternatives would allow insect producers to reduce production costs and ensure the sustainability of their businesses. While most economic models of insect production are centralized, the BSF industry presents a strong potential for decentralization [86]. This would enable on-farm insect rearing facilities to manage waste directly on site (i.e., before spreading), preventing the dissemination of antibiotics in the environment.

The production of antibiotic-medicated feeds for livestock by mills generates each year an unknown quantity of antibiotic residues. These residues originate from sanitary protocols (flush), or products removed from the market because of expiration, batch errors, etc. Given that these organic residues contain variable and often unknown types and levels of antibiotics, they cannot be redirected to animal feed. These residues present a significant potential for upcycling through black soldier flies. To successfully incorporate antibiotic-medicated feeds into livestock feed substrates, it is crucial to investigate the effectiveness of larvae in antibiotic bioremediation, while ensuring the microbiological quality of the final livestock products and maintaining the bioconversion performance essential for industrial livestock production.

Even if the ability of the black soldier fly to do the bioremediation of antibiotic compounds seems promising, the biosecurity of the final products needs to be demonstrated. As covered in this review, the degradation and biodegradation of antibiotic compounds are intimately linked with the environmental conditions and matrix. Therefore, as long as no standard production guidelines are established, each production will have to carefully demonstrate the efficiency and security of bioremediation in their production.

## 8. Conclusions

Antibiotic use in Canadian animal production is a complex issue with far-reaching implications for human health, food security, animal welfare and environmental sustainability. This review presents how veterinary antibiotics use intersects with multiple fields (i.e., agriculture, animal health, human health, environment, policies and new technologies). We aimed to quickly address each of these factors cohesively to show how they impact the utilization and the risks associated with antibiotic use in livestock. While the focus of this review was on veterinary antibiotics in livestock, other sources of antibiotic use, such as pets and humans, also have a significant impact and must be considered within the One Health perspective. The Canadian usage of veterinary antibiotics was reported; however, these datasets have several limitations, as outlined in Section 4. Notably, antibiotics considered not medically important in humans (category IV) are not reported. While the existing system aims to mitigate the risks associated with antibiotic use, primarily through regulatory policies, there is still a significant lack of information to provide a comprehensive picture of the situation in Quebec. Although data is available on a national scale, the vast size of the territory and the diversity of animal production and regulations make such a portrait less relevant at the provincial level. Nevertheless, it appears that the accidental dissemination of antibiotics and ARGs is common across the country. Addressing this issue requires coordinated actions and, more importantly, the development of new approaches to manage antibiotic-contaminated residues. In this regard, upcycling through edible insects is worth investigating and could prove an effective, environmentally sensitive, and economically viable solution to antibiotic residue management. The Canadian flexible regulatory landscape presents a fertile ground for the development of BSF technology, particularly in its application to the bioremediation of antibiotic-contaminated residues. The regulatory adaptability of Canada facilitates innovation in waste valorization and circular bioeconomy initiatives, while maintaining rigorous assessment of safety, creating a conducive environment for the responsible integration of new sustainable agricultural management practices such as the BSF technology.

## Figures and Tables

**Table 1:** Classes of antimicrobials reported to be compounded for use in Canadian livestock [36].

Antimicrobial Class	Antimicrobial Agent
**Aminoglycosides**	Amikacin, apramycin, dihydrostreptomycin, framycetin sulfate, gentamicin, neomycin, spectinomycin, streptomycin
**β-Lactams/Penicillins**	Amoxicillin, ampicillin, cloxacillin, penicillin, sulbactam, clavulanic acid
**Cephalosporins**	Ceftiofur, cephapirin, cefovecin, cefaclor, cefadroxil
**Fluoroquinolones**	Ciprofloxacin, danofloxacin, enrofloxacin, marbofloxacin, orbifloxacin, pradofloxacin
**Synthetic Anticoccidials and Arsenicals**	Amprolium, clopidol, decoquinate, diclazuril, narasin, nicarbazine, pyrimethamine, robenidine, toltrazuril, zoalene
**Ionophore Anticoccidials**	Lasalocid, maduramycin, monensin, salinomycin
**Lincosamides**	Clindamycin, lincomycin, pirlimycin
**Macrolides**	Erythromycin, gamithromycin, tilmicosin, tylosin, tulathromycin, tildipirosine, tylvalosin
**Other Antimicrobials**	Avilamycin, bacitracin, bambermycin, chloramphenicol, chlorhexidine gluconate, florfenicol, fusidic acid, nitarsone, nitrofurantoin, nitrofurazone, novobiocin, polymyxin, tiamulin, virginiamycin
**Tetracyclines**	Chlortetracycline, oxytetracycline, tetracycline
**Trimethoprim and Sulfonamides**	Ormetoprim, sulfabenzamide, sulfacetamide, sulfadiazine, sulfadimethoxine, sulfadoxine, sulfguanidine, sulfamerazine, sulfamethazine, sulfanilamide, sulfaquinoxaline, sulfathiazole, trimethoprim

**Table 2:** Categorization of antimicrobial drugs based on importance in human medicine [48].

Category of Importance	Antimicrobial Class
Category I: Very high importance	Carbapenems, cephalosporins (third and fourth generations), fluoroquinolones, glycopeptides, glycylcyclines, ketolides, lipopeptides, monobactams, nitroimidazoles (metronidazole), oxazolidinones, penicillin-β-lactamase inhibitor combinations, polymyxins (colistin), therapeutic agents for tuberculosis (e.g., ethambutol, isoniazid, pyrazinamide and rifampin)
Category II: High importance	Aminoglycosides (except topical agents), cephalosporins (first and second generations, including cephamycins), fusidic acid, lincosamides, macrolides, penicillins, quinolones (except fluoroquinolones), streptogramins, trimethoprim/sulfamethoxazole
Category III: Medium importance	Aminocyclitols, aminoglycosides (topical agents), bacitracins, fosfomycin, nitrofurans, phenicols, sulphonamides, tetracyclines, trimethoprim
Category IV: Low importance	Flavophospholipids, ionophores

**Table 3:** Antibiotics listed in the Canadian Compendium of Medicating Ingredient Brochures (MIB).

Antibiotic	Class	MIB	Inhibition or Disruption Target	Treatment and Prevention Approved Claim(s)	Concentration in Complete Feed (mg/kg)	Approved Livestock Species	Caution
Avilamycin	Streptogramine	AVI	Protein synthesis	Necrotic enteritis	15–30	Broiler chickens	NA
				Post-weaning diarrhea	80	Swine	
Bacitracin (methylenedisalicylate)	Polypeptide	BACN-M	Cell wall synthesis	Necrotic enteritis	110	Laying hens	NA
				Necrotic enteritis	110	Broiler chickens	
				Clostridial enteritis	275	Pregnant and lactating sows and gilts	
Bacitracin zinc	Polypeptide	BACN_Z	Cell wall synthesis	Reduction in early mortality	110	Chicks	NA
				Necrotic enteritis	55	Broiler chickens	
				Bacterial enteritis	55–110	Swine	
Bambermycin (flavomycin)	Ionophore	BAM	Cell wall synthesis	Increased rate of weight gain and improved feed efficiency	2	Broiler chickens	NA
				Increased rate of weight gain	2	Broiler turkeys	
Chlortetracycline hydrochloride	Tetracycline	CTC	Protein synthesis	Hexamitiasis and synovitis	55–220	Turkeys	NA
				Bacterial enteritis and porcine proliferative enteropathy	55–220	Swine	
				Foot rot	0.22 *	Beef and non-lactating dairy cattle	
				Bacterial diarrhea	55	Calves	
				Reduction in losses due to Enterotoxemia	22	Lambs	
Chlortetracycline hydrochloride, Sulfamethazine, and Penicillin	Tetracycline, sulfamide, penicillin	CSP	Multiaction	Bacterial enteritis	110 CTC, 110 sulfamethazine, 55 penicillin	Swine	NA
Decoquinate	Hydroquinolone	DEC	Electron transport and sporozoite development	Caecal and intestinal coccidiosis	30	Broiler chickens	NA
				Coccidiosis	0.5 *	Cattle and calves	
				Coccidiosis	0.5 *	Lambs	
Florfenicol	Phénicolé	FLOR	RNA synthesis	Furunculosis	200–2000	Salmonids	NA
Lasalocide sodique	Ionophore	LAS	Ionic homeostasis, leading to osmotic lysis	Coccidiosis	105	Broiler chickens	Do not allow horses or other equines access to feeds containing lincomycin, as ingestion may be fatal.
				Coccidiosis	100	Turkeys	
				Increased rate of weight gain	36	Cattle	
				Coccidiosis	36	Calves	
				Coccidiosis	36	Lambs	
Lincomycin	Licosamide	LINC	Protein synthesis	Mycoplasmal pneumonia and porcine proliferative enteropathy, swine dysentery, and disease following treatment	220	Swine	Not for breeding swine. Do not allow rabbits, hamsters, guinea pigs, horses, dairy cattle, or other ruminants access to feeds containing lincomycin.
Monensin sodium	Ionophore	MOS	Protein synthesis	Coccidiosis	100	Broiler chickens and turkeys	Do not allow dogs, horses, other equines, or guinea fowl access to formulations containing monensin. Ingestion of monensin by these species has been fatal.
				Coccidiosis and improve feed efficiency and increased rate of weight gain	22–48	Beef cattle	
				Coccidiosis	11–22	Sheep	
				Coccidiosis	11–22	Goats	
Narasin	Ionophore	NAR	Ionic homeostasis, cellular function, and metabolism	Coccidiosis	70	Broiler chickens	Do not allow canines, horses, or other equines access to formulations containing narasin. Ingestion of narasin by these species has been fatal.
				Increased rate of weight gain and improved feed efficiency	15	Swine	
Oxytetracycline hydrochloride	Tetracycline	OTC	Protein synthesis	Infectious sinusitis and synovitis	110	Turkeys	Do not administer to lactating dairy cattle.
				Bacterial enteritis and abortion caused by leptospirosis	55–550	Swine	
				Bloat in young cattle on pasture and feedlots	75 **	Beef cattle	
				Bacterial enteritis	55	Calves	
				Bacterial enteritis in creep-fed suckling lambs and losses due to enterotoxemia	22–110	Lambs	
				Ulcer disease	75 *	Salmonids	
Penicillin G Procaine	β-lactamines	PEN	Cell wall synthesis	Necrotic enteritis	55	Broiler chickens	Do not feed to laying hens.
Salinomycin sodium	Ionophore	SAL	Signaling pathways by lowering intracellular pH	Coccidiosis	60	Broiler chickens	Do not allow turkeys, dogs, or horses access to this medicated feed, as it is known to be toxic to these species.
				Increased rate of weight gain and feed efficiency	25	Swine	
				Improvement of feed efficiency and growth rate	100 **	Beef cattle	
				Coccidiosis and reduction in coccidian shedding	20	Rabbits	
Sulfadimethoxine and Ormetoprim	Sulfamide and diaminopyrimidine	SMOR	Synthesis of nucleic acids	Furunculosis	5 g of Romet 30 Medicated Premix/100 kg of fish body weight per day	Salmonids	NA
Tiamulin	Pleuromutilines	TIA	Protein synthesis	Swine dysentery, porcine colonic Spirochaetosis, porcine proliferative enteropathy and enzootic pneumonia, and mortality associated with epizootic rabbit enterocolitis	31.2–178.1	Swine	Do not feed animals other than swine.
Tilmicosin	Macrolide	TIL	Protein synthesis	Swine respiratory disease (SRD), porcine polyserositis, and arthritis	200–400	Swine	Do not allow horses or other equines access to feeds containing tilmicosin (toxic for horses).
				Reduction in bovine respiratory disease morbidity	12.5	Feedlot beef cattle	
				Reduction in the severity of respiratory disease	12.5	Rabbits	
Tylosin	Macrolide	TYL	Protein synthesis	Necrotic enteritis	200	Broiler chickens	Do not use in laying hens.
				Cyclic recurrence of swine dysentery, porcine proliferative enteropathy	44–110	Swine	
				Liver abscesses	11	Beef cattle	
Tylvalosin	Macrolide	TYLV	Protein synthesis	Porcine proliferative enteropathy	42.5	Swine	Not for use in breeding animals.
Virginiamycin	Streptogramines	VMY	Protein synthesis	Necrotic enteritis	22	Broiler chickens	Do not feed to birds producing eggs for human consumption, pregnant or lactating females, or animals intended for breeding.
				Swine dysentery	55–110	Swine	
				Liver abscesses	20	Beef cattle	

* mg/kg of body weight per day. ** mg per head by day. NA: Not applicable. https://inspection.canada.ca/en/animal-health/livestock-feeds/medicating-ingredients, accessed on 11 March 2025.

## Data Availability

No new data were created or analyzed in this study. Data sharing does not apply to this article.
